# The role of epigenetic mechanisms in the regulation of gene expression in the cyclical endometrium

**DOI:** 10.1186/s13148-021-01103-8

**Published:** 2021-05-25

**Authors:** Alejandra Monserrat Retis-Resendiz, Ixchel Nayeli González-García, Moisés León-Juárez, Ignacio Camacho-Arroyo, Marco Cerbón, Edgar Ricardo Vázquez-Martínez

**Affiliations:** 1 Unidad de Investigación en Reproducción Humana, Instituto Nacional de Perinatología-Facultad de Química, Universidad Nacional Autónoma de México, Montes Urales 800, Lomas Virreyes, Miguel Hidalgo, 11000 Ciudad de México, Mexico; 2 Departamento de Inmunobioquímica, Instituto Nacional de Perinatología, Ciudad de México, Mexico

**Keywords:** Endometrium, Epigenetic, Proliferative phase, Secretory phase, Sex hormones, Endometrial disease

## Abstract

**Background:**

The human endometrium is a highly dynamic tissue whose function is mainly regulated by the ovarian steroid hormones estradiol and progesterone. The serum levels of these and other hormones are associated with three specific phases that compose the endometrial cycle: menstrual, proliferative, and secretory. Throughout this cycle, the endometrium exhibits different transcriptional networks according to the genes expressed in each phase. Epigenetic mechanisms are crucial in the fine-tuning of gene expression to generate such transcriptional networks. The present review aims to provide an overview of current research focused on the epigenetic mechanisms that regulate gene expression in the cyclical endometrium and discuss the technical and clinical perspectives regarding this topic.

**Main body:**

The main epigenetic mechanisms reported are DNA methylation, histone post-translational modifications, and non-coding RNAs. These epigenetic mechanisms induce the expression of genes associated with transcriptional regulation, endometrial epithelial growth, angiogenesis, and stromal cell proliferation during the proliferative phase. During the secretory phase, epigenetic mechanisms promote the expression of genes associated with hormone response, insulin signaling, decidualization, and embryo implantation. Furthermore, the global content of specific epigenetic modifications and the gene expression of non-coding RNAs and epigenetic modifiers vary according to the menstrual cycle phase. In vitro and cell type-specific studies have demonstrated that epithelial and stromal cells undergo particular epigenetic changes that modulate their transcriptional networks to accomplish their function during decidualization and implantation.

**Conclusion and perspectives:**

Epigenetic mechanisms are emerging as key players in regulating transcriptional networks associated with key processes and functions of the cyclical endometrium. Further studies using next-generation sequencing and single-cell technology are warranted to explore the role of other epigenetic mechanisms in each cell type that composes the endometrium throughout the menstrual cycle. The application of this knowledge will definitively provide essential information to understand the pathological mechanisms of endometrial diseases, such as endometriosis and endometrial cancer, and to identify potential therapeutic targets and improve women’s health.

## Background

The endometrium is one of the most dynamic tissues in the human body as it undergoes periodic changes of regeneration, cell proliferation, differentiation, and apoptosis every 28 days [1]. This tissue is composed of luminal and glandular epithelial cells, stromal cells, immune cells such as lymphocytes and macrophages, endothelial cells, smooth muscle cells, and a recently uncharacterized type of ciliated epithelial cells [2]. The endometrium is one of the most responsive tissues to ovarian steroid hormones. In consequence, the phases of the endometrial cycle coordinate with phases of the ovulation cycle. The terms endometrial cycle and ovulation cycle refer to the menstrual cycle depending on the tissue/organ being studied. The endometrial cycle can be divided into the menstrual phase, the proliferative phase that corresponds to the follicular phase in the ovulation cycle, and the secretory phase that corresponds to the ovarian luteal phase. In turn, the phases of the endometrial cycle are subdivided into early, mid and late proliferative phases and early, mid, and late secretory phases according to histological evaluation [3]. In each menstrual cycle, the endometrium’s functional layer is shed if embryo implantation does not occur and is restored within two weeks [4]. Unless otherwise indicated, from now on we will refer to the menstrual cycle as the endometrial cycle as we will focus on the changes that occur in the endometrium throughout the menstrual cycle.

The average duration of the menstrual cycle is 28 days; nevertheless, it is important to bear in mind that most women experience cycles within a range between 21 and 35 days. The proliferative phase occurs from day 4 to day 14 of the cycle, in which plasma levels of estradiol will increase, reaching their highest levels before ovulation [4]. The increasing amount of estradiol secreted by the ovarian follicles promotes the growth (proliferation) of endometrial glands and stroma; also, there is an increase in the depth of the spiral arteries that supply the endometrium [5]. During this phase, the events that occur include repair of the endometrium surface, proliferation, angiogenesis, vasculogenesis, and extracellular matrix remodeling [4–6].

The secretory phase occurs after ovulation, from day 14 to 28. This phase is characterized by a slight decrease in plasma estradiol levels and a dramatic increase in progesterone levels, leading to a high progesterone to estradiol ratio. During the secretory phase, the endometrium increases its vascular supply, stimulates more mucous secretion, and stops proliferation [7, 8]. At this phase, the endometrium also undergoes a series of transformations to achieve a receptive state for implantation in a process called decidualization [9]. Decidualization is the progesterone-induced transformation of endometrial stromal cells (ESC) into decidual stromal cells (DSC). During this process, the development of the endometrial glands, energy storage in glycogen, and spiral arteries remodeling are observed [6, 10]. Another important characteristic of this phase is the creation of an appropriate environment for blastocyst implantation [6, 10, 11]. If implantation does not occur, estradiol and progesterone levels decline rapidly at the end of the secretory phase, leading to constriction of the spiral arteries, ischemia, and cell death of the functional layer, which in turn causes the shedding of the endometrium [6–11]

The cellular processes that occur during the endometrial cycle are regulated in part by estrogens and progesterone and are associated with specific transcriptional profiles necessary for the proper function of the endometrium [3]. Importantly, these transcriptional profiles are regulated in part by epigenetic mechanisms [12]. Epigenetics has been classically defined as heritable mitotic and meiotic changes in gene function that cannot be explained by modifications in the DNA sequence [13]. Epigenetic mechanisms regulate gene expression temporally and spatially [14] and are involved in fundamental processes like cellular identity, development, homeostasis, and diseases [15]. Epigenetic processes include DNA methylation, histone post-translational modifications (PTMs), chromatin structure, and non-coding RNAs. Chromatin remodeling is necessary to induce specific transcriptional networks by sex hormones throughout the endometrial cycle [16, 17]. The present review aims to provide an overview of current research focused on the epigenetic mechanisms that regulate the function of the cyclical endometrium and discuss the perspectives regarding this topic. We performed a comprehensive review of the literature available in PubMed written in English up to 2020. We used keywords related to DNA methylation, histone post-translational modifications, long ncRNAs, and micro-RNAs, and their combination with the terms “endometrium” and “menstrual cycle”. The information included in the present study refers to the data obtained from healthy subjects (non-pathological endometrium) unless otherwise indicated.

## Transcriptional changes of coding genes in the endometrium during the endometrial cycle

Over the past 20 years, it has been demonstrated that thousands of coding genes change their expression levels in the endometrium throughout the endometrial cycle [2, 18–26]. Although it has been challenging to obtain reproducible results between different studies due to technical and sample limitations, knowledge about the transcriptional networks that mediate functional changes in the endometrium has considerably increased. For example, a recent study has provided fundamental information about the transcription profile of each cell type that composes the endometrium by single-cell transcriptomic analysis throughout the endometrial cycle [2]. This section summarizes the main findings of selected studies that have reported changes in the expression of coding genes related to endometrial function in different phases of the endometrial cycle. For a comprehensive review of transcriptional changes during the endometrial cycle refer to the reviews of [25, 27]. Note that changes in the expression profile of a particular phase are referred to the other phases unless otherwise indicated.

### Expression of essential genes for endometrial function

Cellular processes carried out during the proliferative phase are mediated in part by the functional products of *IGF-1* and *ESR1* genes, whose expression reaches the highest levels during the late proliferative phase of the endometrial cycle [28, 29]. Insulin-like growth factor 1 (IGF-1) is secreted by stromal cells and binds to its receptor (IGF1R) in the epithelium to activate the phosphoinositide 3-kinase (PI3K)/AKT pathway and promote proliferation [30–32]. In the breast cancer MCF7 cell line, estrogen receptor alpha (ERα) has been shown to induce proliferation through the ERK/MAPK pathway [33]. Moreover, estrogens induce the expression of progesterone receptor (PGR) through ERα, which binds to the regulatory regions of the progesterone receptor gene (*PGR)* to induce its expression in a mouse embryonic hypothalamic cell line [34]. Cellular proliferation and induction of *PGR* expression are also key processes in the endometrium regulated by ERα [29].

During the secretory phase, endometrial epithelial cells enter a hypersecretory state to provide the necessary nutrition for embryo survival [35]. The major changes in gene expression observed during the secretory phase occur around implantation. The implantation is a complex progesterone-dependent event that involves several biological processes that occur in a coordinated fashion in the endometrium, such as cell adhesion, cell growth, differentiation, and signal transduction [23, 35]. In a recent study, progesterone has been shown to regulate pathways related to the inflammatory response, xenobiotic metabolism, cell death, epithelial-mesenchymal transition and estrogen response during the window of implantation [36]. The implantation-associated gene clusters include several genes encoding growth factors, such as *TGF*α and *PlGF*. Transforming growth factor alfa (TGFα) mediates various cellular processes, including proliferation, migration, adhesion, and differentiation. Placental growth factor (PlGF) is necessary for the adhesive capacity of the endometrium epithelium and blastocyst growth [23, 37–39]. Lai et al. have revealed that endomucin, an L-selectin ligand, is downregulated in the early secretory endometrium, which may influence endometrial receptivity [40, 41].

In the menstrual phase, the expression of genes encoding inflammatory cytokines, enzymes involved in eicosanoid biosynthesis, and immunomodulators (and their receptors) is increased compared to the proliferative and secretory phases. Genes encoding angiogenic modulators, hypoxia-induced proteins (such haem oxygenase-1, adrenomedullin, vascular endothelial growth factor (VEGF), cysteine-rich angiogenic inducer 61 (CYR61), and hypoxia-induced protein-1, and matrix metalloproteases are also highly expressed in the menstrual phase [25, 29, 42, 43].

### Endometrial functions revealed by transcriptome analysis

Microarray analysis has shown that some genes, including *TGFB2*, *MT2A, F2RL2*, *PLIN2*, and *CCL18,* are upregulated during the early proliferative phase. These genes are required for the regeneration of the *functionalis* layer of the endometrium after menstruation [44]. During the mid-proliferative phase, genes involved in cell renewal processes such as cell proliferation, cell survival, and regulation of differentiation (such as *IHH, SERP4, PGR, SNRD14E,* and *GSTM1)* are upregulated [44]. During the late proliferative phase, the upregulation of *AGTR2, HMGIC*, *C9orf131*, S*NORA23*, and *CRIM1* genes is associated with cell growth inhibition, extracellular matrix remodeling, and cellular differentiation [45]. Interestingly, Petracco et al. identified low expression levels of genes that are related to natural killer (NK) cells function, such as *KIR2DL3* and *KLRC3,* at the late proliferative phase*,* suggesting a decreased immune response mediated by NK cells at this phase of the endometrial cycle, which is consistent with the modulation of the immune response to favor embryo implantation [44, 46]. Besides, genes involved in tissue remodeling, such as *MMP26* and *TFF3,* cell differentiation, such as H*OXA10* and *HOXA11,* vasculogenesis, such as *GJB6, HOXB7,* and *sFRP,* and angiogenesis, such as *CXCR4, CDH5, ENG*, and *PECAM1,* are upregulated during the proliferative phase [22, 47, 48].

The expression profiles of coding genes have also been studied by next-generation sequencing in the proliferative endometrium [25]. We have performed a functional enrichment analysis of the data from Sigurgeirsson, et al. by the g:Profiler software [49] and the top ontology terms of the overexpressed genes during the proliferative phase (top differentially expressed genes with a fold change > − 2.00 from the original data) are related to extracellular matrix and cell cycle processes. This information is consistent with the above-mentioned studies and the single-cell transcriptomic study in which cell cycling was elevated in unciliated epithelia and stromal fibroblasts [2]. Furthermore, by using single-cell transcriptomic analysis, it has been confirmed that the proliferative phase is divided into two transitional phases and that luminal epithelia and glandular epithelia differ by the enrichment of overexpressed genes during this stage, the former by expressing genes related to the development of anatomic structures and the latter expressing genes related to cell cycle [2].

On the other hand, the coding gene expression profiles of endometrial tissue during the secretory phase have also been studied by next-generation sequencing [19, 25]. By using this approach, genes encoding for solute carrier proteins and genes related to endometrial receptivity, metabolism, negative regulation of cell cycle, among others, were identified as highly expressed genes during the secretory phase. Regarding to the specific changes in the transcriptional profiles of each cell type that conforms the endometrium, it has been demonstrated that there is an abrupt change in the transcriptomic profile in the unciliated epithelia at the window of implantation (overexpressing genes like *PAEP*, *GPX3,* and *CXCL14*). In contrast, stromal cells display a more continuous phase transition (overexpressing genes like *DKK1* and *CRYAB*). Wang. et al. point out that the decidualization process in stromal cells is transcriptionally different when comparing the processes that occur after implantation and without implantation [2].

A comparison between the transcriptional profiles of epithelial and stromal areas microdissected from the human endometrium during the secretory phase has been made by microarray analysis [50, 51]. This analysis demonstrated that 28 genes displayed a differential expression profile between the stromal and epithelial compartments. *WFDC2, MMP7, MSX2, HOXB5, HOXB7*, and *PRKCQ* [51] genes were highly expressed in the epithelial area; these genes are involved in sperm maturation, immune system regulation, cell–cell adhesion, among other functions that are necessary for the secretory endometrium [51–53]. On the other hand, *DCN, DDR2, TIMP1, RPS3A*, and *TIE1* [51] genes were strongly expressed in the stromal areas; these genes are involved in mediating cellular responses to the extracellular matrix and regulation of the immune system [51, 54–56].

Accurate determination of the endometrial phase is a crucial factor in the study of gene expression changes in the endometrium. Over time, researchers have developed different methodologies to identify the endometrial cycle phase, including self-reported endometrial cycle [57], determination of serum hormone levels [58], and histological evaluation of endometrial biopsy [3]. However, the inaccurate determination of the specific endometrial phase and other factors (such as genetic heterogeneity and environmental factors) has caused a great variability in the results obtained between different studies. Recent advances in molecular biology have allowed the use of high-throughput microarray technology to classify endometrial samples according to the global transcriptional profile, which is consistent with the histological evaluation [23, 59].

Sample size is another crucial factor that impacts the variability of the results obtained from transcriptional studies. Overall, most published studies have included small sample sizes mainly due to the invasive endometrial biopsy procedure [23, 59]. Larger sample size numbers (along with painless sampling methods) are needed to avoid bias in the results due to individual variability.

Microarray technology has been used for many years to determine the transcriptome at a specific condition. Although this technology allows the analysis of thousands of genes by using specific probes, it is limited in the detection of new transcripts [59]. The use of next-generation sequencing technologies has overcome this limitation as RNA sequencing can detect novel transcripts and provides other relevant information, such as genomic rearrangements and mutations, with higher specificity and sensitivity [23]. However, both microarray and RNA sequencing technologies assume that every cell present in a particular tissue or condition is homogeneous, regardless of the molecular changes of individual cells. To overcome this limitation, transcriptome analysis by single-cell technology coupled with RNA sequencing enables the detection of RNA molecules in individual cells with the advantages of next-generation sequencing [2].

Further studies using single-cell technologies are necessary to compare the transcriptomic data of the different cell types that integrate normal and pathological endometrium, since only data from whole tissue has been analyzed so far [60, 61]. Taking into account the data obtained from differential expression studies, gene silencing in a particular type of endometrial cells (stromal or epithelial) using CRISPR-Cas9 technology could help to elucidate the relevance of specific genes in the pathogenesis of endometrial diseases, such as endometriosis (the presence of endometrium in an abnormal or ectopic location) and endometrial cancer. CRISPR-Cas9 could also be helpful in clarifying the importance of specific proteins necessary for embryo implantation that are downregulated in the endometrium of women with recurrent miscarriages [62, 63]. This approach will provide new information about the pathological mechanisms of these diseases and new potential therapeutic targets.

## DNA methylation

DNA methylation is the chemical modification of DNA, where a methyl group is transferred to the fifth carbon of the cytosine to generate 5-methylcytosine (5-mC). DNA methylation is catalyzed by a family of DNA methyltransferases (DNMT) [64]. DNMT3a and DNMT3b catalyze de novo DNA methylation, while DNMT1 binds to hemimethylated DNA to maintain the DNA methylation pattern after replication [64, 65]. DNA demethylation is achieved by active enzymatic demethylation or by passive replication-dependent dilution of methylation [65, 66]. In the active enzymatic demethylation, 5-methylcytosine (5mC) undergoes a series of oxidation reactions catalyzed by the methylcytosine dioxygenases Ten Eleven Translocation (TET) enzymes. The oxidative products are excised by thymidine DNA glycosylase and replaced by unmodified cytosines through the base excision repair mechanism [66]. The 5-hydroxymethylcytosine (5hmC) is the best-studied and first intermediate of active DNA demethylation [67].

High levels of DNA methylation in the promoter region of genes are frequently associated with transcriptional silencing [68]. DNA methylation in these regions could directly hinder transcription factor binding [69] or recruit reader proteins that contain a specialized domain that identifies and interpret DNA methylation, known as methyl-binding proteins (MBPs) [70]. MBPs then recruit different members of the chromatin remodeling complex to cause transcriptional repression [69, 70].

The effect of this epigenetic mark depends on the genomic context and the specific cytosines that are modified. For example, most CpG sites are not methylated when located at promoters of expressed genes, whereas CpG sites located in gene bodies are frequently methylated in a tissue-specific manner [14, 68]. DNA methylation in enhancer regions is associated with tissue-specific gene expression regulation [71, 72]. Interestingly, cell type-specific super-enhancers are hypomethylated to induce the expression of genes associated with cell identity [73, 74]. Abnormal enhancer methylation is associated with alterations in gene expression in multiple diseases, including many types of cancer [75]. Besides being an intermediate in active DNA demethylation, enrichment of 5hmc in promoter regions is often associated with activation of gene expression [68].

### DNA methylation and the cyclical endometrium

One of the epigenetic mechanisms that control the endometrium’s cyclical changes is DNA methylation [68, 76, 77]. DNA methylation profile has been correlated with its biological age, and changes in this profile have been associated with abnormal function of the endometrium [78]. The variation in DNA methylation content across the menstrual cycle is tissue-specific as the methylation state of endometrial tissue changes according to the endometrial phase; in contrast, this is not observed in blood samples [79]. This suggests that DNA methylation regulates gene expression in a tissue-specific manner during the endometrial cycle.

**Fig. 1:**
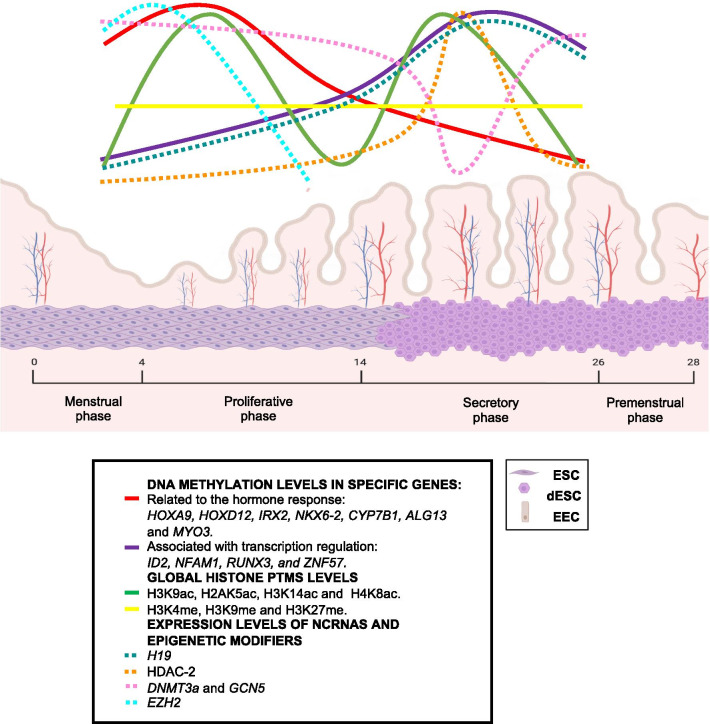
Epigenetics changes in the endometrium throughout the menstrual cycle. The endometrium cycle is divided into the menstrual phase, the proliferative phase, and the secretory phase. The proliferative phase occurs from day 4 to day 14 of the cycle, according to the length of the 28-days menstrual cycle. This phase promotes the growth (proliferation) of endometrial glands and stromal cells (ESC); also, there is an increase in the depth of the spiral arteries that supply the endometrium. The secretory phase occurs after ovulation from day 14 to 28. During this phase, the endometrium’s proliferation is stopped as this tissue undergoes a series of transformations to achieve a receptive state for implantation in a process called decidualization. Decidualization is the transformation of ESC into decidual stromal cells (DSC) induced by progesterone. If no implantation occurs, constriction of the spiral arteries producing ischemia and cell death of the functional layer causes the endometrium to shed, and this phase is denominated menstrual phase. During the proliferative phase, there is an increase in the DNA methylation levels of hormone-responsive genes and the expression of *DNMT3a*, *GCN5,* and *EZH2* genes. On the other hand, during the secretory phase, an increase in the DNA methylation levels of genes associated with transcription regulation, the protein content of HDAC-2, and the expression of *H19* is observed. The global content of H3K9ac, H2AK5ac, H3K14ac and, H4K8ac is downregulated during the ovulation and menstrual phase. No changes have been reported for the global levels of H3K4me, H3K9me, and H3K27me. Endometrial epithelial cells (EEC)

The content of DNA methylation in the human endometrium varies across the endometrial cycle [76, 80, 81]. Differential methylation states between the proliferative and secretory phases are found in genes associated with transcriptional regulation, cell proliferation, and regulation of RNA metabolic processes, regardless of whether they contain CpG islands (CGI) [76]. For example, genes that do not have CGI, like *ADORA1, C21orf128,* and *TRPV3,* are more methylated in the proliferative phase than in the secretory phase [76]. Genes related to the hormone response like *HOXA9, HOXD12, IRX2, NKX6-2, CYP7B1, ALG13, MYO3A,* and *FZD2* are less methylated in the secretory phase than in the proliferative phase; meanwhile, genes associated with transcription regulation, e.g., *ID2, NFAM1, RUNX3,* and *ZNF57* are more methylated in the secretory phase [82] (Fig. 1). In addition, genes associated with embryo implantation like *MAPK14*, *ZMIZ1,* and *PLXNA4* are less methylated during the receptive phase than the pre-receptive phase of the endometrial cycle [83]. Interestingly, the DNA methylation status of several differentially methylated genes correlated with their expression levels as reported in other tissues [76, 84, 85]. This suggests that the dynamics of DNA methylation in the endometrium are regulated in part by the hormone milieu, which can influence the function of this tissue by modulating its transcriptome. Recent studies have identified several endometrial phase-dependent changes in the DNA methylation content of genes regulated by estrogens or progesterone throughout the endometrial cycle [79, 86]. Moreover, estradiol, progesterone, or the combination of both modifies the DNA methylome of primary cultures of stromal cells in a hormone-specific manner, with estradiol inducing the most extensive changes [86].

### DNA methylation machinery in the cyclical endometrium

**Fig. 2:**
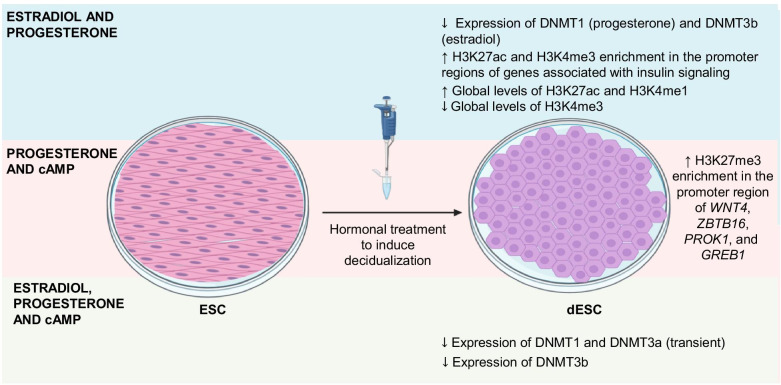
In vitro decidualization and the regulation of epigenetic processes. On the left side of the figure are shown the different methodologies used to induce in vitro decidualization. On the right side of the figure are shown the effects of in vitro decidualization on the content of epigenetic processes and components. Estradiol and progesterone downregulate the expression of DNMT1 (mainly by progesterone) and DNMT3b (mainly by estradiol), increase the enrichment H3K27ac and H3K4me3 in the promoter regions of genes associated with insulin signaling, increase the global content of H3K27ac and H3K4me1, and decrease the global content of H3K4me3. Progesterone and cAMP increase the enrichment of H3K27me3 in the promoter region of *WNT4*, *ZBTB16*, *PROK1*, and *GREB1* genes. Estradiol, progesterone, and cAMP downregulate the expression of DNMT3b and transiently decrease the expression of DNMT1 and DNMT3a

The expression of DNMTs in the human endometrium is differentially regulated across the endometrial cycle and by in vitro hormonal treatments. *DNMT3a* expression is downregulated during the secretory phase and under estrogens and progesterone treatments in endometrial explant cultures (Fig. 1), whereas *DNMT1* and *DNMT3b* expression is downregulated only by one of the hormones, progesterone and estrogens, respectively [87] (Fig. 2). Besides estradiol and progesterone, cAMP induces the decidualization process. In an immortalized endometrial stromal cell line, the expression of DNTM3b is downregulated by the action of estradiol, medroxyprogesterone (MPA), and cAMP, whereas DNMT1 and DNMT3a are transiently downregulated [88] (Fig. 2). It has been recently reported that estradiol induces the expression of DNMT1 and DNMT3b in cell cultures of immortalized stromal and epithelial endometrial cells, respectively, while estradiol and progesterone decrease the expression of DNMT1 in the stromal cells [89].

The relationship between steroid hormones and DNA methylation in the endometrium remains unclear, whether the hormone stimuli is sufficient to activate DNMTs and induce specific methylation patterns in the genome that, in consequence, would lead to a particular gene expression profile, or DNA methylation is the mechanism that controls the action of these hormones. In other models such as the brain [90], breast cancer [91], and CD4 + cells [92], steroid hormones have been shown to regulate DNTMs activity, DNA methylation, and gene expression [92]. Regarding the female reproductive system of other mammals, estrogens and progesterone inhibit *dnmt3a* and *dnmt3b* expression in Siberian hamsters’ uterus [93]. The relation between sex hormones and DNMTs can be speculated; nevertheless, further investigation is warranted to elucidate the crosstalk among steroid hormones, progesterone and estradiol, and DNA methylation in the cyclical human endometrium.

Another important component of the DNA methylation machinery are the MBD protein family (readers) and TET enzymes (erasers). The MBD protein family includes several members, namely MeCP2, MBD1, MBD2, MBD3, and MBD4 [94]. The expression of *MBD2* is higher in the secretory phase compared to the proliferative and menstrual phases and under estradiol and progesterone treatments in endometrial explant cultures, whereas MBD1 and MeCP2 expression is not modified throughout the endometrial cycle nor by hormonal treatments [95]. On the other hand, the expression of *TET1* and *TET3* is higher in the mid-secretory phase than in the other phases of the endometrial cycle. In vitro treatments with progesterone induce *TET1*, *TET2,* and *TET3* expression in endometrial epithelial cells and estradiol plus progesterone treatments increase the expression of *TET3* in the same cell type, while estradiol induces the expression of *TET1* in stromal cells. Interestingly, all three TET proteins were detected in both epithelium and stroma throughout the endometrial cycle [89]. These studies suggest that sex hormones regulate the expression of *MBD2* and *TET* genes in a dynamic and cell-specific manner in the human endometrium. The role of the DNA methylation readers and erasers in the endometrium has not been elucidated, and further studies are required to establish their participation in the regulation of gene expression throughout the endometrial cycle.

### DNA methylation in the window of implantation

In the secretory phase, the endometrium undergoes multiple changes that makes it more receptive to the blastocyst in case of implantation [10]. Several changes in DNA methylation content are observed during the implantation window as precise regulation of gene expression is required for the implantation and establishment of the blastocyst. It has been proposed that any alteration in the DNA methylation profile of the receptive endometrium would lead to aberrations in gene expression, which in turn would impede the decidualization process and embryo implantation [96, 97]. In fact, alterations in the DNA methylation content have been associated with defective receptivity. Particularly, 448 differentially methylated CpGs have been found between women who have recurrent pregnancy failure and healthy women, which was associated with a possibly altered immune response and G protein activity due to their involvement in endometrial receptivity [98].

*HOXA* genes are essential for embryo implantation. Increased DNA methylation levels have been found in the promoter region of *HOXA10* and *HOXA11* genes in the endometrium of women with repeated implantation failure compared to control women [99]. Interestingly, hypermethylation of *HOXA* genes is reversed by the demethylating agent 5-aza-2’-deoxycytidine (5-AZA) in Ishikawa endometrial cancer cells, which in turn increased the expression of *HOXA10* and its target genes, *ITGB3* and *IGFBP1,* which play an important role in endometrial receptivity [100].

High progesterone levels at the time of ovulation have been proposed to modulate the methylation status of genes necessary for implantation [1]. Particularly, high progesterone levels induced by human chorionic gonadotropin (hCG) administration increase the global content of DNA methylation and DNMT3B expression in endometrial epithelial cells [101, 102]. This increase in DNA methylation was associated with hypermethylation of the promoter regions of *CDH1* and *CNTNNB1* genes and the consequent decrease in gene expression [102]. These genes encode the adhesion molecules required in the implantation process, suggesting a possible explanation of IVF treatment’s failure, at least in part, by DNA methylation. Superovulation induced by gonadotropins administration is another condition that creates an altered hormonal milieu, in which changes in DNA methylation has been associated with an increase in the expression of genes related to endometrial remodeling such as *PLAT, MMP2,* and *TIMP1* and the downregulation of *HSPE2* that could alter trophoblast migration and impair endovascular invasion [103]. These studies set a precedent about the effects of exogenous hormone administration on the content of DNA methylation in the endometrium, especially at the time of ovulation, in women who undergo an IVF procedure as these molecules could modify certain epigenetic marks needed for implantation in different endometrial cell types. Moreover, it is imperative to consider the impact of exogenous hormone treatments in the studies assessing the role of epigenetic modifications in endometrial function as these treatments may induce several molecular and cellular effects, including the regulation of DNA methylation.

The studies described above indicate that DNA methylation is influenced by the hormone milieu at the window of implantation, especially by progesterone, and alterations in the hormone levels could lead to a different DNA methylation pattern that could disturb the implantation process. These findings highlight the importance of studying differentially methylated genes and the specific methylation site in all the cell types present in the endometrium and different hormonal contexts, probably by single-cell DNA methylation profiling.

Most studies have focused on identifying changes DNA methylation levels in the promoter region of genes; however, as mentioned previously, DNA methylation in enhancer regions has an important role in regulating gene expression associated with cell identity [71, 72]. Therefore, future research is required to focus on studying DNA methylation in these regulatory regions as well.

The lack of evidence of the direct effect of DNA methylation on gene expression is an important limitation of these studies as the methylation status of a particular CpG site could affect the expression and function of several other DNA elements in addition to the promoter of the closest gene [14]. The next step in this research field would be a gene-specific modification of the DNA methylation status with a CRISPR/Cas9 related system to validate its effect on gene expression [104, 105]. Furthermore, modulation of DNA methylation at specific CpG sites or a single-base mutation within a CpG (associated with a methylation quantitative trait loci) could be performed using this technology to assess the effects on gene expression.

### DNA methylation in endometrial pathologies

DNA methylation changes in the female reproductive system are linked to disease [84]. Particularly, DNA methylation is altered in specific genomic regions in endometrial pathologies, and differences in the content of this epigenetic modification are influenced by the particular phase of the endometrial cycle [82, 85]. Single nucleotide polymorphisms (SNPs) are changes in DNA sequence that occur in a single nucleotide and are present in at least 1% of the population. SNPs located within a CpG may impact DNA methylation, challenging the classical definition of epigenetics [106–109].

In a study conducted by Mortlock et al. [79], a methylation quantitative trait loci (mQTL) analysis was performed to identify the relationship between SNPs associated with pathologies of the endometrium (e.g., endometriosis) and DNA methylation. In this study, an association between specific SNPs and DNA methylation was found in blood samples and endometrium, suggesting mQTLs as potential biomarkers for endometrial pathologies. This study established a paradigm where although blood and endometrium have a different cellular composition, an association between SNPs and DNA methylation was found in both samples. This finding suggests that blood samples are a less invasive alternative to studying the DNA methylation changes in healthy and pathological endometrium. However, more studies are needed to confirm this association as differences in the content of DNA methylation between proliferative and secretory phases were found in the endometrium but not in blood. This study also highlights the difficulty to determine whether the differences in DNA methylation observed in blood and endometrial tissue are associated with the diverse cellular population of both samples, the local concentration of hormones, or both. These limitations should be addressed by single-cell technologies to obtain the DNA methylation profile of specific cell types in a particular phase of the endometrial cycle.

Differences in the methylation status of specific genes have been found between the early and late development stages of endometrial cancer, especially in genes encoding transcription factors [110]. TETs and MBD proteins, such as MECP2, have been implicated in endometrial cancer and endometriosis [95, 111–113]. More research is warranted to assess the relationship between DNA methylation, DNA methylation machinery, hormonal status, and endometrial diseases such as endometriosis and endometrial cancer, which would allow the discovery of more precise biomarkers and new potential therapeutic targets.

## Histone post-translational modifications

The eukaryotic gene expression would be senseless without considering the landscape in which it occurs, the chromatin. The chromatin is formed by the nucleosome consisting of 146-147 bp of DNA wrapped around a histone octamer, which is made by two copies of each inner histone, namely H2A, H2B, H3, and H4 [114]. The amino acids in histone N-terminal tails interact with the DNA and are also a target of PTMs. These PTMs are acetylation, methylation, phosphorylation, ubiquitination, SUMOylation, citrullination, and ribosylation, among others [115]. These modifications constitute the histone code. The histone code is a hypothesis that assumes that different PTMs act together in a coordinated manner to influence chromatin conformation [116]. Such modifications are reversible and catalyzed by enzymes that deposit them (writers) and remove them (erasers).

These modifications regulate gene expression through two mechanisms. The first mechanism is based on electrostatic interactions, since DNA is negatively charged and histone N-terminal tails are positively charged. For example, acetylation of a lysine residue will change the positive charge to a negative one. The negative charge will generate a more relaxed chromatin state; if this occurs in the promoter region, transcription factors and RNA polymerase II (Pol II) have more room to bind DNA and activate transcription [108]. Histone acetylation is catalyzed by histone acetyltransferases (HATs) and is removed by histone deacetylases (HDACs) [117–119]. The second mechanism by which PTMs regulate gene expression requires specific proteins, called readers, that interpret the modification. This type of PTM creates a binding site for transcription factors, chromatin remodeler complexes such as chromatin remodelers of the switch/sucrose-non-fermenting (SWI/SNF), imitation switch (ISWI), chromodomain-helicase-DNA binding (CHD), and inositol requiring 80 (INO80) families, and adaptor proteins that, in turn, regulate transcription [120, 121].

Chromatin immunoprecipitation assays followed by microarrays (ChIP-chip) and next-generation sequencing (ChIP-seq) have provided fundamental information about the role of a variety of histone modifications and their localization in the genome [121, 122]*.* Promoters of transcriptionally active genes are enriched with histone H3 lysine 4 trimethylation (H3K4me3) and histone H3 and H4 lysine acetylation, while in gene bodies, they tend to have higher levels of trimethylation of histone H3 lysine 36 (H3K36me3) and trimethylation of histone H3 lysine 79 (H3K79me3) [123]. After transcription initiation, histone H3 lysine 9 acetylation (H3K9ac) is necessary to induce the release of Pol II pausing by directly recruiting the super elongation complex (SEC) to chromatin [124]. The enrichment of H3K9ac in the genome is highly correlated with that of histone H3 lysine 14 acetylation (H3K14ac), and the enrichment levels of both are also correlated with other histone marks (such as H3K4me3), suggesting a coordinated enrichment of active histone marks. Acetylation of histone H4 at lysine 8 (H4K8ac) is among the most dynamic histone PTMs preferentially occupying the 5′ intergenic regions (5′IGRs) and 5′ termini of the open reading frames (ORFs) of several genes [125]. Enrichment of histone H3 lysine 16 acetylation (H3K16ac) and Pol II occupancy are observed in actively expressed regions in the genome [126].

On the other hand, histone H3 lysine 27 trimethylation (H3K27me3) and histone H3 lysine 9 trimethylation (H3K9me3) are known for their relationship with transcriptionally repressed chromatin in metazoan genomes. Several protein complexes catalyze these modifications, including the Enhancer of Zeste Homolog 2 (EZH2), which is the catalytic subunit of the Polycomb Repressive Complex 2 (PRC2) and several H3K9 methyltransferases [127].

### Histone post-translational modifications and the cyclical endometrium

Another of the epigenetic mechanisms that control the transcriptional changes throughout the endometrial cycle is the histone PTMs (Fig. 1). Global acetylation of H3 and H4K8 is modulated by ovarian steroid hormones, estradiol and progesterone, in ESC [128]. Ligand-activated hormone receptors, such as PR and ERα, act by binding to the proximal promoters and distal regulatory elements of target genes to recruit components of the basal transcription machinery and cofactors, such as HATs and HMTs, that induce the histone PTMs and alter the chromatin structure [17, 129, 130].

The global levels of histone acetylation in the endometrium vary during the endometrial cycle. Particularly, global levels of H3K9ac, H2A lysine 5 acetylation (H2AK5ac), H3K14ac, and H4K8ac are increased in the early proliferative phase, subsequently declining in the late proliferative phase [12]. This increase in the global acetylation of histones could be associated with the activation of many genes and pathways necessary for the regeneration of the endometrium’s functional layer, such as endometrial epithelial growth, angiogenesis, and proliferation pathways [131–133]. However, more studies are required to prove this statement. An increase in the global acetylation levels is also observed after ovulation, which could be involved in the progesterone-dependent transcriptional activation of secretory associated pathways required to differentiate endometrial glands and stroma [12]. The tight regulation of gene expression during the secretory phase is critical to induce a specific network for ESC decidualization, which is a prerequisite for successful implantation [134]. During the late secretory phase, the global levels of these histone PTMs decline, suggesting that the decrease in hormone levels (estradiol and progesterone) resulting from the regression of the corpus luteum could be involved in the loss of global acetylation [12]. In contrast to these findings, Monteiro et al*.* did not observe significant differences among the H3K9ac and H3K16ac throughout the endometrial cycle, probably because Munro et al. evaluated the levels of PTMs in the subphases of the cycle, while Monteiro et al. only study the changes in PTMs at proliferative versus secretory endometrium [12, 135] (Fig. 1).

Class I HDACs and HATs are differentially expressed in the human endometrium during the endometrial cycle, which may explain the differences in the content of acetylated histones [136]. Particularly, the content of HDAC-2 protein increases in the secretory phase in parallel with reduced levels of GCN5, which is a HAT (Fig. 1). These findings suggests a shift in the balance between histone deacetylation and acetylation towards deacetylation, which may explain the changes in the global level of this PTM during the late secretory phase [136]. Interestingly, Uchida et al*.* reported that histone deacetylase inhibitors (HDACI) could induce changes in morphology, gene expression, differentiation, and function in endometrial epithelial and stromal cells [137]. Further studies are warranted to establish the role of several HATs and HDACs in each subphase of the endometrial cycle and their associated transcriptional programs.

**Fig. 3:**
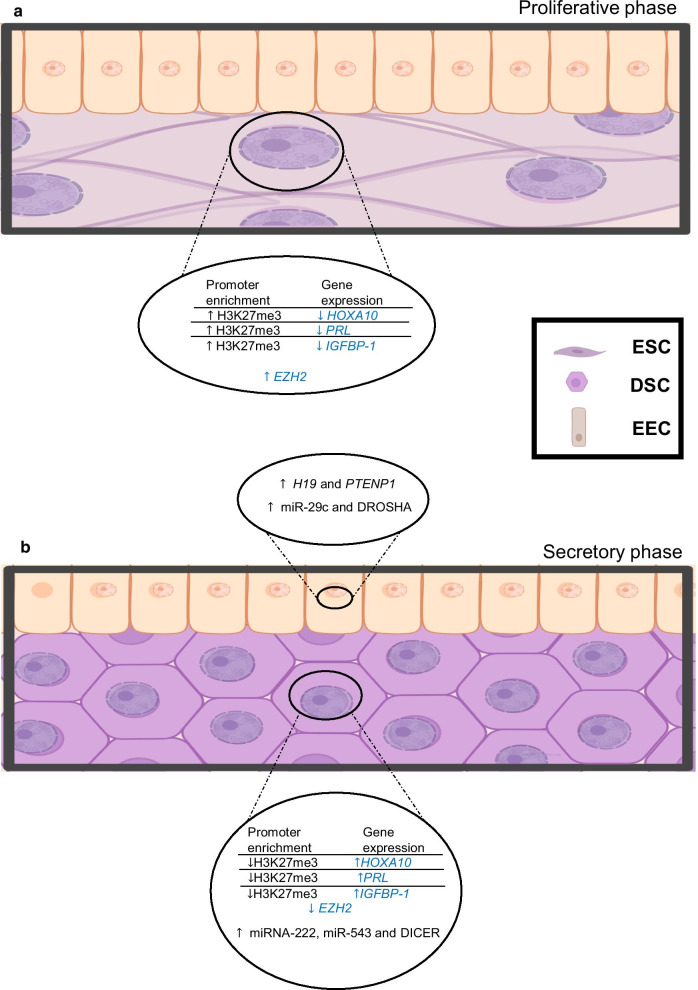
Epigenetics changes during the proliferative and secretory phases in endometrial cells. a. During the proliferative phase, the enrichment of H3K27me3 on the *HOXA10, PRL,* and *IGFBP-1* promoters in endometrial stromal cells (ESC) is reported, which is consistent with the lack of expression of these genes. Also, an increase in the expression of *EZH2*, which catalyzes the H3K27me3 modification, is observed. b. A decrease in the enrichment of H3K27me3 in the *HOXA10, PRL,* and *IGFBP-1* promoters in decidual stromal cells (DSC) during the secretory phase is reported; consequently, an increase in the expression of these genes is observed. Furthermore, a decrease in the expression of *EZH2* during decidualization is observed in DSC. During the secretory phase, some miRNAs, lncRNAs, and the proteins involved in their biogenesis increase their expression compared to the proliferative phase, such as miRNA-222, miR-543, and DICER in DSC, and miR-29c, DROSHA, *H19,* and *PTENP1* in endometrial epithelial cells (EEC)

On the other hand, it has been reported that global levels of histone methylation of H3K4, H3K9, and H3K27 do not change across the endometrial cycle [135] (Fig. 1), perhaps because these study only looked at proliferative versus secretory endometrium and did not consider the same subphases of the cycle as analyzed by Munro et al*.* [12, 135]*.* However, specific changes in the content of these histone PTMs have been found in particular genes. For example, it has been reported that the enrichment of H3K27me3 on the *HOXA10* promoter is higher in the proliferative phase in comparison with the secretory phase [138] (Fig. 3a). HOXA10 is a homeodomain transcription factor essential for normal uterine embryogenesis and endometrial cycle regulation. The enrichment of H3K27me3 in *HOXA10* promoter is consistent with previous studies showing that *HOXA10* is dynamically expressed in the endometrium, which is downregulated during the proliferative phase and highly expressed in the secretory phase [138, 139] (Fig. 3b).

### Impact of in vitro decidualization in the enrichment of histone post-translational modifications

The expression of EZH2, which catalyzes H3K27 trimethylation, is decreased during in vitro decidualization and the transition of proliferative to secretory phase. The downregulation of EZH2 activity in the endometrium during decidualization is correlated with the loss of H3K27me3 enrichment in *PRL* and *IGFBP-1* genes (key decidual marker genes induced by progesterone), suggesting that H3K27me3 is an integral part of the chromatin remodeling process that enables the transition from a proliferative to a decidual phenotype in response to different signals such as progesterone and cAMP [140] (Fig. 3). Furthermore, the subsets of up- and downregulated genes upon decidualization are associated with reciprocal changes in the enrichment of histone H3 lysine 27 acetylation (H3K27ac) and H3K27me3 modifications, respectively, at their promoter region. Essential genes for the decidualization of ESC such as *WNT4*, *ZBTB16*, *PROK1*, and *GREB1* contain high levels of H3K27ac in their promoter after the treatment with 8-Bromo-cAMP and progesterone, confirming the dynamic properties of chromatin in response to decidualization [16, 140] (Fig. 2). Interestingly, cAMP alone cannot induce an increase in the enrichment of H3K27ac in the regulatory regions of *PRL* and *IGFBP-1* genes in ESC, suggesting that these epigenetic changes are stimulus and gene-specific [141].

Tamura et al. found several genomic regions enriched with H3K27ac after ESC decidualization. Interestingly, 80% of these regions were located in distal gene regions, which may function as enhancer elements to induce the expression of decidualization associated genes. Pathway analysis revealed that upregulated genes enriched with H3K27ac or H3K4me3 modifications were associated with the insulin signaling pathway, which may be involved in glucose uptake necessary for decidualization [142] (Fig. 2). It has been demonstrated that decidualization induces the enrichment of H3K27ac in the distal upstream region (-4701 to -7501 bp) of the *IGFBP-1* promoter [143]. Furthermore, the transcriptional regulators CCAAT enhancer-binding protein β (C/EBPβ), FOXO1, and p300 binds to this potential enhancer to induce *IGFBP-1* expression [143]

Recently, the transcriptional and chromatin landscapes of cultured ESC collected from human placental membranes, and the corresponding DSC (induced by MPA and cAMP treatments) have been characterized. A total of 1,135 differentially expressed genes were identified after decidualization, of which upregulated genes were associated with insulin-related terms and glucose metabolism, while downregulated genes were associated with cell cycle [142, 144, 145]. Interestingly, the enrichment of H3K27ac and H3K4 monomethylation (H3K4me1) was higher in DSC than ESC in several genomic regions, and the opposite pattern was observed for H3K4me3, which was more enriched in ESC than DSC (Fig. 2). These findings indicate that the epigenetic changes underlying gene expression alterations during decidualization predominantly occur in potential enhancers [145]. Additionally, the evaluation of the accessible chromatin regions by ATAC-seq revealed tens of thousands of differentially accessible regions between ESC and DSC. Genomic regulatory regions with more accessible chromatin overlap with the enrichment of H3K27ac and H3K4me1 enhancer marks in upregulated genes. Conversely, the potential enhancer regions of downregulated genes became less accessible after decidualization [145].

Taken together, all these findings indicate that histone PTMs are important players in gene expression regulation in the endometrium during the endometrial cycle. However, the specific role of ovarian steroid hormones on the regulation of histone modifiers activity throughout the endometrial cycle remains to be elucidated. More studies using next-generation sequencing are necessary to establish the distribution of several histone PTMs in the endometrium during the different subphases of the endometrial cycle and in specific endometrial cell types by single-cell approaches [146]. Applying these technologies in in vitro models of decidualization will help to identify the role of chromatin structure generated in response to steroid hormones. In addition, global and locus-specific changes in the content of particular histone PTMs have been reported in endometriosis and endometrial cancer [135, 138, 140, 147–151]. However, more studies are required to establish the biological function of each histone PTM in these diseases, which will help in the discovery of new potential therapeutic targets as in other types of cancer or diseases.

## Non-coding RNAs

Non-coding RNAs (ncRNAs) are defined as functional RNA molecules without protein-coding ability and are divided into small RNAs (containing less than 200 nucleotides) that include small interfering RNAs (siRNAs), microRNAs (miRNAs), and PIWI-interacting RNAs (piRNAs), and long ncRNAs (lncRNAs) that contain more than 200 nucleotides [152]. NcRNAs interact with nuclear proteins such as histone-remodeling complexes or DNMTs to regulate gene expression. In addition, lncRNAs and miRNAs regulate gene expression by directly interacting with other RNA molecules such as mRNAs [153]. In this section, we are focus on lncRNAs and miRNAs. LncRNAs can function as a) decoys that titrate away DNA and RNA-binding proteins; b) scaffolds to bring proteins into complex or spatial proximity; c) a guide to recruiting proteins to the DNA; and d) an enhancer (eRNA), to create loops that bring DNA regions together through a scaffolding complex [154, 155]. On the other hand, miRNAs are small molecules of 18–22 nucleotides that post-transcriptionally regulate gene expression by the binding to the 3’-end of the target mRNA, and more rarely to the 5’-end, via the RNA-induced silencing complex, leading to the inhibition of mRNA translation by different mechanisms [156].

Recent studies have shown that enhancers function as transcriptional units and are transcribed to produce eRNAs, which are also involved in the regulation of gene transcription [157, 158]. Despite the growing evidence supporting eRNAs as functional biomolecules [159–161], the mechanism by which eRNAs promote gene transcription remains unknown. However, eRNAs can bind to transcription factors, influence RNA Pol II elongation, and mediate enhancer-promoter interactions [162].

Circular RNAs (circRNA) are endogenous ncRNAs that range in size from 100 nucleotides to more than 4 kilobases. These molecules are produced by back-splicing, wherein a downstream 5′ splice site (splice donor) is joined to an upstream 3′ splice site (splice acceptor) [163]. CircRNAs act as protein sponges, interact with RNA-binding proteins (RBPs), enhance protein function, and recruit proteins to specific locations [164–168].

### Non-coding RNAs and the cyclical endometrium

It has been recently demonstrated that RNA molecules have an interplay with each other creating the competitive endogenous RNA (ceRNA) network, in which several RNA molecules regulate gene expression and several biological functions; this intricate interplay typically involves lncRNA-miRNA and mRNA molecules [169, 170]. In the endometrium, ceRNA networks have been shown to regulate the implantation process, the dynamics between trophoblast and endometrium during pregnancy, and have also been used as biomarkers for disease prognosis in endometrial cancer and to identify which pathways are regulated by lncRNA-miRNA competition [171–176]. During the implantation window, the regulation of mRNA levels by lncRNA-miRNA molecules in the endometrium is critical. This is the case for *H19*/let-7a-5p/*ITGB3* where *H19* acts as a sponge for let-7a-5b to prevent the degradation of *ITGB3* transcript that is translated into a protein necessary for implantation [174, 177]. This association between lncRNAs with other RNAs to create ceRNA networks could be involved in many functions needed to prepare the endometrium for embryo implantation, such as immune system activity, angiogenesis, apoptosis, and steroid biosynthesis [178].

### lncRNAs

The transformation of the endometrium throughout the endometrial cycle is mainly driven by the steroid hormones, progesterone and estradiol, which can influence the expression profile of ncRNAs [179]. However, the expression of ncRNAs in the normal cyclical endometrium has not been studied in-depth as most studies focus on endometrial pathologies and implantation failure, which are discussed below.

One of the most studied lncRNAs is the product of the *H19* imprinted gene, whose expression in normal endometrium fluctuates during the endometrial cycle and has only been detected in the endometrial stroma. *H19* reaches its highest expression during the late secretory phase when progesterone is the dominant hormone, suggesting that this gene could be regulated by sex hormones [180, 181] (Fig. 1). *H19* is reciprocally imprinted with respect to *IGF2*, regulating *IGF2* imprinting and expression [182]. Interestingly, *IGF2* is upregulated during the decidualization process [183]; however, the relation between *H19* and *IGF2* during decidualization remains to be explored. *H19* is also expressed in endometrial cancer tissues [184]. The knockdown of this lncRNA in endometrial cancer is associated with reduced migration, invasiveness, and tumor growth [184, 185]. In the endometrium of women with endometriosis, decreased *H19* expression is associated with decreased endometrial stromal cell proliferation as this lncRNA reduces the availability of miRNA let-7, which inhibits *IGF1R* expression that is necessary for cell proliferation and proper endometrial function [174]. In addition, the expression of *H19* is downregulated in the secretory endometrium of patients with repetitive implantation failure (RIF) compared to the control group. This differential expression could lead to the downregulation of integrin β-3 necessary for trophoblast adhesion [174, 177, 186]. Furthermore, the differential expression of lncRNAs in patients with RIF has been associated with a dysregulation of pathways necessary for a proper implantation process, such as cell adhesion, tumor necrosis factor signaling, Toll-like receptor signaling, and NK-κB signaling [187, 188]. These studies also suggest that progesterone, the main regulator of the decidualization process, could be regulating the expression of *H19* and *IGF2* to accomplish implantation [180–182].

The expression of many lncRNAs is upregulated during decidualization, such as *NEAT1*, *RP11-627G23.1*, and *PSORS1C3*, but their function in this process remains to be elucidated [25]. These lncRNAs may regulate the expression of genes associated with cell proliferation, differentiation, migration, and angiogenesis during decidualization, as proposed in mice uteri [189]. *PTENP1*, a lncRNA that is highly expressed in the luminal epithelium during the mid-secretory phase, regulates the expression of several miRNAs to promote the implantation process [173] (Fig. 3b). It has been suggested lncRNA *H2KP1,* along with its associated gene *HK2,* is necessary for endometrial decidualization as it inhibits cell proliferation [190]. Interestingly, specific pathways regulate the expression of lncRNAs required for decidualization, such as the cAMP-PKA pathway that regulates *LINC00473* expression, whose specific function in the decidualization process is still unknown [191].

Another lncRNA, *ENST00000433673*, is expressed in epithelial and stromal endometrial cells to enhance the synthesis of ITGAL and ICAM1, two proteins required for the adhesion of the trophoblast to the endometrium [192]. LncRNAs have also been proposed as biomarkers of endometrial receptivity, such as *LINC01060* and *LINC01104*, whose specific functions remain to be elucidated [193].

### miRNAs

The expression of miRNAs during the endometrial cycle has been evaluated in endometrial and serum samples. Even though there is evidence that miRNAs levels in serum do not change throughout the menstrual cycle and an endometrial biopsy is recommended to determine the endometrial phase in terms of expression profiles, there is one study that suggests the use of miRNAs as serum biomarkers to determine the receptivity of the endometrium during the secretory phase [194, 195]. Throughout the endometrial cycle, progesterone and estradiol regulate the expression of different miRNAs in the endometrium, and these miRNAs regulate several genes involved in extracellular matrix remodeling, cell proliferation, and the response to steroid hormones [196, 197]. In addition, the administration of progesterone and estradiol in assisted reproduction cycles modulates miRNA profiles that may regulate endometrial receptivity [196].

The secretory phase shows more changes in the gene expression profiles of the endometrium compared to the menstrual and proliferative phases. These changes in gene expression are partly mediated by miRNAs that target cell cycle related-genes to suppress cell proliferation [18]. The different cell types that compose the endometrium express different miRNAs related to endometrial receptivity during the secretory phase. In stromal cells, miR-543 expression increases during the secretory phase and has been suggested to be involved in signaling pathways associated with endometrial receptivity [198] (Fig. 3b). During decidualization, increased expression of miRNA-222 is associated with a decrease in the number of stromal cells in the S phase of the cell cycle, while high levels of miR-29c expression in epithelial cells are associated with the proper attachment of the blastocyst to the endometrium [199, 200] (Fig. 3b). These studies highlight the importance of miRNAs function in the different cell types that are part of the endometrium.

Another aspect that should be considered in the study of the cyclical endometrium is the regulation of miRNA biogenesis. Particularly, the highest expression of DROSHA (responsible for the initiation of miRNAs processing) is observed in the epithelium during the early and mid-secretory phases, whereas DICER (responsible for the processing of miRNA precursors into mature miRNAs) is preferentially localized and highly expressed in the stroma during the late secretory phase (Fig. 3b). The content of these proteins is lower in the endometrium of infertile women than the endometrium of fertile women during the same phase of the endometrial cycle, which could indicate that these proteins are important for endometrial function [201]. The specific role of these ribonucleases in the cyclical endometrium should be explored in future studies.

Specific miRNA families, such as the miRNA-30 family, or single miRNAs, modulate the expression of genes involved in the regulation of implantation [202, 203]. Particularly, several miRNAs have been associated with implantation failure, for example, miR-29c and miR-661 that reduce the content of collagen type IV alpha 1 (COL4A1) and the E3 Ubiquitin Protein Ligase (MDM2), respectively; both proteins are necessary for the adhesion of the trophoblast to the endometrial epithelial cells [200, 204, 205]. During the first weeks of pregnancy, the decidualized endometrium shows a specific miRNA expression profile. Particularly, miRNA-146b-5p, miRNA-181b-5p, miRNA-424, miRNA-532, and miRNA-199a-3p are downregulated, while miR-423, miR-22-3p, let-7i-5p, and miR-1 are upregulated in the decidualized endometrium from early pregnancy compared to proliferative endometrium; these miRNAs are associated with genes that are necessary for the maintenance of pregnancy [206]. miRNAs have also been proposed as potential biomarkers for assessing endometrial receptivity because of their relationship with coding-gene expression, which may help to identify the appropriate time for embryo implantation [207–209]. For example, miR-22 downregulates Tiam1/Rac1 protein levels required for embryo implantation in mice. Interestingly, the expression of these molecules is regulated by estradiol and progesterone, and it is proposed that they are involved in RIF [210].

### eRNAs

Little is known about the role of eRNAs in the cyclical endometrium. It has been reported that the expression of the eRNA long noncoding-CES1-1 (*lnc-CES1-1*) is upregulated in decidual tissue from patients with unexplained recurrent pregnancy loss (URPL) compared to controls. The transcription factor signal transduction and activation of transcription 4 (STAT4) induces the expression of *lnc-CES1-1* in decidual associated cell lines. In turn, this associated eRNA binds to the fused in sarcoma (FUS) transcription factor to induce *PPARγ* expression, inhibit cell migration and increase the inflammatory response, which may explain its association with URPL [211].

In the Ishikawa endometrial adenocarcinoma cell line, estradiol induces the expression of an eRNA transcribed from an enhancer that induces the expression of *GREB1* [212]. Interestingly, *GREB1* is highly expressed in the endometrium, and its expression levels fluctuate in accordance with estrogen levels throughout the endometrial cycle in healthy women [213]. However, the role of eRNAs in the regulation of *GREB1* expression in the cyclical endometrium remains to be elucidated.

### circRNAs

To the best of our knowledge, only one study has explored the expression profile of circRNAs in the endometrium, in which a total of 21,340 circRNAs were differentially expressed between endometrial cancer and healthy endometrium [214]. Nevertheless, the expression and function of circRNAs in the cyclical endometrium are unknown.

More transcriptomic studies are needed to investigate the expression profiles of lncRNAs, miRNAs, eRNAs, and circRNAs in the endometrium during the proliferative, secretory and menstrual phases, as well as those regulated by sex hormones, which in turn could be responsible for the regulation of expression of coding genes in each endometrial phase. In addition, little is known about how these RNA molecules could interact with each other during the implantation process and how the hormone milieu could influence the expression of the ncRNA molecules to support the endometrium for implantation.

The differential expression of lncRNAs and miRNAs has been associated with diverse pathologies, and their study has helped to elucidate the molecular mechanisms involved in the pathogenesis of different diseases. In some cases, ncRNAs have also been used as prognosis biomarkers for disease [215–219]. In pathologies of the endometrium, e.g., endometrial cancer and endometriosis, the study of ncRNAs has not been the exception as the study of these molecules has contributed to clarifying the molecular mechanisms that rule these diseases [217, 220, 221]. Considering that the best method for diagnosing these diseases is laparoscopy surgery, it is of great interest to find more informative biomarkers that can be identified in plasma or serum samples to avoid invasive procedures and improve the life quality of patients [217, 222, 223]. Moreover, a complete understanding of the role of these molecules in endometrial diseases may lead to the discovery of potential therapeutic targets. Further studies are required to establish the action mechanisms of lncRNAs, since they can mediate long-range DNA interactions to regulate gene expression of several genes involved in endometrial cancer pathogenesis [224]. In addition, the participation of other ncRNAs, such as eRNAs and circRNAs, during the different phases of the endometrial cycle and endometrium disease should be explored.

## Conclusion and perspectives

Epigenetic mechanisms are emerging as key players in regulating transcriptional networks associated with fundamental processes and functions of the cyclical endometrium. Particularly, DNA methylation, histone PTMs, and ncRNAs regulate the expression of genes associated with endometrial epithelial growth, angiogenesis, and stromal cell proliferation during the proliferative phase. During the secretory phase, these epigenetic mechanisms promote the expression of genes associated with hormone response, insulin signaling, decidualization, and embryo implantation. Further studies using next-generation sequencing are warranted to explore the role of other covalent modifications of nucleic acids and histones, such as DNA hydroxymethylation, RNA methylation, and novel histones PTMs, as well as other non-coding RNAs that are being recognized as fundamental factors in regulating gene expression such as eRNAs or circRNAs. It is clear now that single-cell studies will elucidate the role of the epigenetic mechanisms in each cell type that composes the endometrium, as recently demonstrated by the single-cell transcriptomic atlas reported by Wang *et* al. [2].

The human genome has a complex and hierarchical structure, called the tridimensional organization of the genome, which plays a fundamental role in regulating gene expression [225, 226]. The genome folds into different levels of organization, including chromosomal territories, open (named A) and closed (named B) chromatin compartments, autonomously folded substructures called topologically associated domains (TADs), and interactions between two specific genomic regions that create chromatin loops [227]. In this regard, a recent study identified a total of 53,211 interactions between promoters and distal regions with accessible chromatin in decidua-derived ESCs decidualized with MPA and cAMP during 48 h [145]. These findings suggested that these interactions should have a regulatory role in the decidualization process. However, more studies are required to explore the functional role of these interactions and the role of the tridimensional organization of the genome to create functional TADs in the nucleus of endometrial cells throughout the endometrial cycle.

Finally, aberrant epigenetic changes may contribute to the pathogenesis of reproductive diseases such as endometrial cancer or endometriosis. Epigenetic mechanisms are probably also dysregulated in women with RIF, thus preventing the correct transition of proliferative endometrium to a decidualized environment necessary in case of fertilization. The application of this knowledge will definitively provide essential information to understand the pathological mechanism of endometrial diseases, such as endometriosis, RIF, and endometrial cancer, and to identify potential therapeutic targets.

## Declarations

## Data Availability

All the information is included in this manuscript.

## Abbreviations

5-mC: 5-Methylcytosine
5′IGRs: 5′ Intergenic regions
5hmc: 5-Hydroxymethylation
ATAC-seq: Assay for Transposase-Accessible Chromatin with high throughput sequencing
bp: Base pair
cAMP: 3',5'-Cyclic adenosine monophosphate
circRNA: Circular RNAs
ceRNA: Competitive endogenous RNA
CGI: CpG islands
ChIP-chip: Chromatin immunoprecipitation assay followed by microarrays
ChIP-seq: Chromatin immunoprecipitation assay followed by next-generation sequencing
CRISPR/Cas9: Clustered regularly interspaced short palindromic repeats-Cas9
CYR61: Cysteine-rich angiogenic inducer 61
DNA: Deoxyribonucleic acid
DNMT: DNA methyltransferases
DSC: Decidual stromal cells
EEC: Endometrial epithelial cells
eQTL: Expression quantitative trait loci
ERα: Estrogen receptor alpha
eRNAs: Enhancer RNAs
ESC: Endometrial stromal cells
EZH2: Enhancer of Zeste Homolog 2
H2AK5ac: Acetylation on histone H2A lysine 5
H3K14ac: Acetylation on histone H3 lysine 14
H3K16ac: Acetylation on histone H3 lysine 16
H3K27ac: Acetylation on histone H3 lysine 27
H3K27me3: Trimethylation on histone H3 lysine 27
H3K36me3: Trimethylation on histone H3 lysine 36
H3K4me1: Monomethylation on histone H3 lysine 4
H3K4me3: Trimethylation on histone H3 lysine 4
H3K79me3: Trimethylation on histone H3 lysine 79
H3K9ac: Acetylation on histone H3 lysine 9
H3K9me3: Trimethylation on histone H3 lysine 9
H4K8ac: Acetylation on histone H4 lysine 8
HATs: Histone acetyltransferases
hCG: Human chorionic gonadotropin
HDACI: Histone deacetylase inhibitors
HDACs: Histone deacetylases
IVF: In vitro fertilization
lncRNAs: Long ncRNAs
miRNAs: MicroRNAs
MPA: Medroxyprogesterone
mQTLs: Methylation quantitative trait loci
mRNA: Messenger RNA
ncRNAs: Non-coding RNAs
ORFs: 5′ Termini of the open reading frames
piRNAs: PIWI-interacting RNAs
Pol II: RNA polymerase II
PR: Progesterone receptor
PRC2: Polycomb Repressive Complex 2
PTMs: Post-translational modifications
RNA: Ribonucleic acid
SEC: Super elongation complex
siRNAs: Small interfering RNAs
SNPs: Single nucleotide polymorphisms
TADs: Topologically associated domains
TNFα: Transforming growth factor alfa
TSSs: Transcription start sites
VEGF: Vascular endothelial growth factor
URPL: Unexplained recurrent pregnancy loss
